# A study of notation dependence in fraction magnitude processing using event related potentials

**DOI:** 10.1038/s41598-025-23957-1

**Published:** 2025-11-17

**Authors:** Weimin Lin, Yun Pan, Jun Zhu, Liangzhi Jia, Huanyu Yang, Yajie Bi, Fangwen Yu, Di Zhang

**Affiliations:** 1 School of Psychology, Guizhou Normal University, Guiyang, China; 2 Guizhou Education University, Guiyang, China

**Keywords:** Number cognition, Symbolic and non-symbolic fractions, Notation dependent, Distance effect, ERP, Mathematics and computing, Neuroscience, Psychology, Psychology

## Abstract

Increasingly, research emphasizes that learning symbolic fractions (i.e., the quotient or ratio of two whole numbers) is challenging for both children and adults. However, humans and certain non-human animals are capable of representing the proportional relationships between non-symbolic magnitudes (e.g., dot arrays presented in different colors). The specific mechanisms underlying the relationship between symbolic and non-symbolic magnitude processing are still not sufficiently clear. In this study, we employed event-related potential (ERP) technology to investigate the relationship between symbolic and non-symbolic fractions in numerical processing. We found that, compared to symbolic fractions, the N1 component amplitude evoked by non-symbolic fractions was significantly more negative. When participants processed non-symbolic fractions, there was a significant difference in P3 amplitude between far distances and close distances; however, such differences were not observed when processing symbolic fractions. These findings lend support to the notion of a representation of fractions that is dependent on notation. This suggests that the processing of symbolic and non-symbolic fractions involves two distinct numerical systems.

## Introduction

 Fractions are commonly encountered in our daily lives, such as in the measurement of half a cup of water or one-quarter of a pizza. The concept of fractions constitutes a significant mathematical construct that follows the concept of integers. Unlike integers, which represent numerical magnitudes in a discrete manner, fractions provide ordered and continuous representations, offering greater precision than integers^1,2^. For instance, the fraction “2/5” illustrates a proportional relationship by expressing the quotient or ratio of the two integer components “2” and “5”. The understanding of fractions constitutes a crucial component of mathematics education and is an indispensable element within the mathematics curriculum. The study of fractions represents a crucial transition in individuals’ understanding of numbers, extending from integers to rational numbers. This foundational knowledge lays the groundwork for the subsequent acquisition of more complex mathematical concepts^3,4^. The significance of understanding and applying fractions is self-evident, as fractions are widely regarded as the foundation of mathematical achievement. The understanding of fractions may serve as a significant predictor of children’s future algebraic skills and their general mathematical performance^5–10^. For the representation of fractions, in addition to symbolic fractions, there also exist non-symbolic fractions (e.g., two line segments of differing lengths). Non-symbolic proportion refers to the representation of the proportional relationship between two non-symbolic magnitudes. Such representation is based on the Approximate Number System (ANS)^11,12^. From the early stages of human development, individuals possess the ability to process non-symbolic magnitudes^11,13–17^. For instance, prior to formal schooling, six-month-old infants have already demonstrated the capacity to distinguish between ratios of two different magnitudes^11^. Humans, non-human primates, and other species possess the ability to process large magnitudes of numerical information^16,18–20^. Some non-human animals are not only capable of representing individual non-symbolic magnitudes, but also of representing the proportional relationships between two non-symbolic magnitudes^21–25^. For instance, Drucker et al. (2016) conducted an experiment on the judgment of the proportion of rhesus macaques. The results showed that rhesus macaques could compare arrays of discrete items based on the proportion of items in each array^21^. Before the acquisition of language, individuals are capable of naturally obtaining non-symbolic numerical ratio information. However, for both children and adults, learning symbolic fractions presents a significant challenge. Current research indicates that there are inconsistent findings regarding the neural basis of symbolic and non-symbolic number processing, which has sparked significant controversy in this field^26–32^. Therefore, it is worthwhile to explore whether there are similar debates regarding the cognitive neural foundations of symbolic fractions and non-symbolic fractions.

### Theoretical background and related work

The study of symbolic and non-symbolic magnitudes is a significant direction in the field of numerical cognition. While there has been extensive research on both non-symbolic and symbolic magnitudes, the findings from related studies are not entirely consistent. Some studies suggest that symbolic magnitudes and non-symbolic magnitudes are processed using the same mechanism^32–35^. A substantial body of behavioral research has demonstrated the existence of the distance effect in both studies concerning symbolic numbers and those addressing non-symbolic numbers^33,34,36–38^. Furthermore, research that combines symbolic and non-symbolic numbers has similarly demonstrated the presence of the ratio effect^34,35,38–40^. The distance effect reflects the processing of numerical information^41,42^. Specifically, when participants are required to compare two values with a large absolute difference, their performance is typically superior to that observed when comparing values with a smaller absolute difference. There is also neurological evidence indicating that the mechanisms for processing numerical and non-numerical symbols are shared, existing within the same neural representations^32,35,43^. For a long time, it has been widely believed that the processing of symbolic numbers relies on the processing of non-symbolic numbers^44–46^. The ANS mapping theory posits that, in the process of handling magnitudes of symbols, individuals instinctively map the magnitude of symbols to the corresponding non-symbolic magnitudes^47^. Previous research indicates that neuroimaging studies focused on numerical magnitude consistently identify the intraparietal sulcus (IPS) as a key brain region responsible for numerical processing^32,46,48–51^. For instance, Holloway and Ansari (2010) conducted a functional magnetic resonance imaging (fMRI) study in which both children and adults performed numerical comparison tasks involving symbolic (Hindu-Arabic numerals) and non-symbolic (arrays of squares) representations, along with two control tasks. The findings revealed significant activation in the right IPS^52^. Ansari et al. (2006) employed a passive viewing paradigm in which participants were required to observe slides that rapidly changed and contained arrays of squares. The findings indicated that the IPS is involved to some extent in the differentiation of numerical magnitudes^48^. As research on digital processing and brain imaging continues to advance, an increasing body of evidence suggests that the bilateral prefrontal cortex plays a crucial role in numerical processing^53–56^. Arsalidou and Taylor (2011) conducted a meta-analysis examining the brain regions associated with basic numerical processing and calculation tasks in adults. Their findings support the notion that the frontal cortex plays a important role in numerical processing among adults^57^. Mussolin et al. (2013) conducted a fMRI study involving children aged 8 to 14 years, who participated in a comparison task of Arabic numeral pairs as well as a control task comparing non-numeric symbols by color. The results indicated significant activation in the frontal lobe regions^54^. Additionally, there is corresponding evidence from ERP research. Libertus et al. (2007) conducted a electrophysiological study in which participants performed numerical comparison tasks involving both symbolic and non-symbolic representations. The results indicated that the numerical distance effect under non-symbolic conditions was consistent with that observed under symbolic conditions; specifically, the amplitude of the P2p component was significantly greater when close to the standard value compared to when it was far from the standard value^58^. In summary, numerical processing takes place in a manner that is independent of notation. This indicates that the neural processing patterns associated with both symbolic and non-symbolic numerical manipulation are identical.

However, some studies have proposed an alternative perspective, suggesting that the processing mechanisms for symbolic and non-symbolic magnitudes differ and that these two processes operate independently of one another^26,43,59–63^. Under varying conditions—namely symbolic, non-symbolic, or mixed conditions—the ratio exerts differing effects on the outcomes. Vanbinst et al. (2012) conducted a study examining the relationship between numerical magnitude representation and arithmetic operations, revealing that children’s symbolic number processing abilities, rather than their non-symbolic number processing skills, are associated with individual differences in arithmetic performance^62^. Sasanguie et al. (2017) conducted two experiments utilizing an audiovisual matching paradigm to investigate the relationship between symbolic and non-symbolic number processing. The findings revealed that, unlike in non-symbolic tasks, no ratio effect was observed in symbolic number tasks^43^. This discovery suggests that there may be two distinct numerical systems underlying performance in these tasks. Herrera and Macizo (2008) conducted a study on symbolic semantic priming and found that the priming effect occurred only in the sequence from dot arrays to numbers, while no such effect was observed in the reverse order^64^. This finding indicates that numerical processing is influenced by the format of the digits.

Previous neuroimaging studies have also supported the concept of notation dependence^26,65–69^. Vogel et al. (2015) conducted a study utilizing passive functional magnetic resonance imaging with an adaptive design to investigate the representation of symbolic numerical magnitude in the cerebral cortex of children aged 6 to 14 years. The results indicated that the left posterior parietal sulcus is generally considered to be involved in the processing of symbolic numbers. Furthermore, there exists a hemispheric difference in the involvement of the medial parietal cortex during the developmental process of symbolic number representation^69^. Bulthé et al. (2014) investigated the neural representations of symbolic numbers (digits) and non-symbolic numbers (dot arrays) through multivoxel pattern analysis (MVPA). The findings revealed that there is no overlap in neural representations between symbolic and non-symbolic numbers across three different spatial scales. Furthermore, relevant neurophysiological research findings indicate that individuals exhibit differences in processing symbolic ratios compared to non-symbolic ratios^26^. Jay (2019) conducted an ERP experiment to investigate the temporal processing of symbolic and non-symbolic ratio information by individuals. The results indicated that when ratio information was presented in a non-symbolic format, a distance effect was observed in the P3 component; however, no such effect was found for symbolic ratios^70^. The coding complexity hypothesis proposed by Campbell et al. posits that non-symbolic and symbolic magnitudes are notion dependent^71–73^. According to this theory, numbers are not represented in an abstract manner; instead, they rely on independent modality-specific numerical coding. Therefore, based on the above discussion, there exist two distinct magnitude representation systems for symbolic and non-symbolic numerical processing.

### Related work on symbolic and non-symbolic fractions

The aforementioned discussion indicates that there remains considerable controversy regarding the processing mechanisms of symbolic and non-symbolic magnitudes. The research on symbolic and non-symbolic numerical processing has primarily focused on the domain of integers in the past. The integrated theory of numerical development posits that the development of integers is interconnected with that of fractions, suggesting that advancements in integer understanding can predict developments in fraction comprehension^3,4^. The processing of symbolic fractions involves not only holistic representation strategies (i.e., considering the actual numerical value of the overall fraction) but also component-based representation approaches (i.e., inferring the magnitude of a fraction based on the sizes of the numerator and denominator). Specifically, the processing of symbolic fractions may be influenced by interference from whole number strategies^74,75^. For instance, in the study conducted by Bonato et al. (2007)^76^, participants were asked to compare different target fractions (e.g., 1/4, 1/7) with a standard value (1/5). The findings indicated that fraction processing occurs in a componential manner, as only the absolute distance between the denominators of the target fractions and that of the standard value significantly influenced response times. Liu et al. (2013) carried out a study investigating how children represent fractions that share common components. The findings revealed that all children utilized mental number lines to represent the fractions, and they employed a component-based representation strategy when comparing fractions with identical numerators^77^. Previous research has indicated that during the process of fraction processing, individuals select appropriate processing strategies based on the requirements of the task and the characteristics of the stimuli^78,79^. Meert et al. (2010a) employed a priming paradigm to examine children’s responses to fraction representations. The findings revealed a significant increase in reaction times (RTs) when participants were presented with pairs of fractions that shared the same numerator but differed in their denominators (e.g., 4/7 and 4/5). This effect was particularly pronounced when the denominator was larger (e.g., 7)^80^. Gabriel et al. (2013) conducted a study on the same/different judgment task regarding fractions, employing two distinct tasks: one was the physical matching task, and the other was the numerical matching task. The results showed that in the numerical matching task, the overall value of the fractions could be obtained. However, in the physical matching task, the participants relied on the component strategy^81^. These studies indicate that when processing fractions, participants employ both componential and holistic processing strategies. Regarding non-symbolic fractions, some researchers have proposed that the representation of non-symbolic ratios is not grounded in the ANS, but rather relies on a specialized system for representing and processing ratio information known as the Ratio Processing System (RPS)^82–84^. Based on observed differences in counting between integers and fractions, these researchers argue that while ANS may provide a cognitive foundation for the symbolic representation of integers, it may be insufficient as a cognitive basis for fraction representation. Recent studies have emerged that explore the relationship between symbolic fraction abilities and non-symbolic proportional reasoning skills^85–88^. Specifically, Lv et al. (2023) found that these findings indicate a correlation between non-symbolic and symbolic fraction abilities during development; however, this association appears to weaken over time^86^. Starling-Alves et al. (2022) found that mathematical anxiety has a negative impact on symbolic fraction skills, while it does not affect non-symbolic mathematical skills^88^. Therefore, the relationship between non-symbolic fraction ability and symbolic fraction ability remains unclear.

### The current study

Similar to whole numbers, the study of the processing of symbolic fractions and non-symbolic fraction has also been subject to similar controversies. Currently, there is relatively limited research in this area, and existing studies lack clarity. Compared to traditional behavioral experiments, neuroimaging techniques such as fMRI and electroencephalography (EEG) may demonstrate greater sensitivity in detecting complex cognitive mechanisms^89,90^. The temporal resolution of fMRI technology is relatively low, which limits its ability to fully reveal the real-time processing of numerical information and associated neural activities. The ERP technique entails the measurement of the brain’s electrophysiological responses that are time-locked to specific events, such as sensory, cognitive, or motor activities. This approach facilitates the analysis of neural cognitive processes with a temporal resolution on the order of milliseconds. Unlike fMRI technology, ERP techniques exhibit superior temporal resolution, allowing for the precise detection of potential differences within brief time intervals across varying conditions^90^. Therefore, this study employs ERP technology in conjunction with distance effects to investigate the dynamic changes occurring in the brain between symbolic fractions and non-symbolic fractions. The distance effect refers to the phenomenon whereby a decrease in the numerical distance between comparative values leads to an increase in reaction time (RT) and a corresponding decline in accuracy^91^. The numerical distance effect has been extensively validated through numerous studies in the field of numerical cognition^58,92–95^.

Based on previous research on integers, the N1 component reflects the processing of symbolic stimuli discrimination, while the P3 component represents the activation of numerical magnitude representation^55,58,92,95,96^. For the P3 component and distance effects, it is commonly observed that larger numerical differences or easier classifications are associated with greater amplitudes of the P3 component^92,96,97^. Here, we refer to the research paradigm established by Jay (2019)^70^to explore the symbolic specificity in the processing of non-symbolic and symbolic fractions. Research has indicated that there are differences in cognitive processing between continuous representation and discretization or discrete representation^74,98–100^. Specifically, Abreu-Mendoza et al. (2023) conducted a study that revealed the relationship between nonsymbolic and symbolic proportional skills may be influenced by misconceptions arising from discretized representations, rather than by an understanding of proportional magnitudes^74^. Therefore, in this study, we employed a proportion composed of discrete non-numeric stimuli (such as dot arrays), which differs from the approaches used in previous research. We examine whether ratios composed of discrete non-numeric stimuli (dot arrays) produce findings comparable to those reported in prior research^70^ that utilized vertical lines as stimuli. If a similar outcome can be achieved through ratios generated by discrete stimuli, this would provide strong support for the stability of non-symbolic fractions in numerical representation. More importantly, this study seeks to explore the distinctions between the processing of non-symbolic and symbolic fraction magnitudes. We hypothesize that if the symbolic format effect on the N1 component is significant, it indicates the presence of notation-dependent during the symbol discrimination phase; If the P3 component exhibits an interaction between symbolic format and distance effects, this indicates that the numerical representation stage possesses notation-dependent. Conversely, if the opposite result occurs, it indicates that the processing of ratio magnitudes is not influenced by surface forms and exhibits notation-independent.

## Materials and methods

### Participants

The sample size was estimated using G*Power 3.1. The parameters were set as follows: f = 0.25, number of measurements = 4, α = 0.05, power = 0.80, and correlation among repeated measures = 0.50, which indicates that a total of 24 participants should be recruited^101^. A total of 32 participants were initially recruited for the study. However, due to excessive EEG artifacts caused by head movements, 3 participants were excluded from the analysis. Consequently, a final sample of 29 participants remained for further examination. In the actual data collection of neurophysiological experiments such as EEG, data are often discarded due to excessive signal artifacts or other reasons. We adopted a conservative approach and actively recruited more participants than the minimum requirement. A larger sample size can further reduce the risk of Type II errors, enabling us to have greater confidence in detecting the truly existing effects and ensuring that the statistical power is higher than the conventional standards adopted in psychological research. In a sample of 29 participants, there were 16 females. The age range of the participants was between 18 and 22 years, with an average age of 20.24 years (SD = 1.46). All participants were right-handed and had no history of mental illness or neurosis. Their vision was either normal or corrected to a normal level. Furthermore, this study has received approval from the Ethics Committee of the College of Psychology at Guizhou Normal University. All participants signed an informed consent form prior to the commencement of the experiment. After the conclusion of the experiment, participants will receive a specified compensation.

### Materials and equipment

The stimulus materials consist of a randomly arranged white and black dot arrays, with each dot array corresponding to a specific fraction. The number of white dots serves as the numerator, while the number of black dots functions as the denominator. These visual stimuli were presented at the center of the computer screen, with all dot arrays restricted to a virtual circular area having a diameter of 6° of visual angle. The diameters of the individual dots vary randomly between 26 and 55 pixels, while ensuring that the distance between any two dots is no less than 16 pixels. All points are randomly distributed within the virtual circular ring, and it is ensured that the cumulative area of each point in different dot arrays remains equal. The brightness value of the grey background (RGB: 190, 190, 190) is 60 cd/m^2^, the brightness value of the white dot is 220 cd/m^2^, the brightness value of the black dot is 0.3 cd/m^2^, the contrast between the white dot and the background is 0.57, and the contrast between the black dot and the background is 0.99. The randomization process was completed by the E-Prime 2.0 (Version 2.0.10; https://pstnet.com/products/e-prime).

According to previous research^70^, we categorize the distance between fractions into two levels: close distance and far distance. Distance is defined as the ratio of the smaller fraction to the larger fraction. When this ratio is less than 0.325, it is considered a far distance; conversely, when it exceeds 0.62, it is regarded as a close distance. Select the fractions based on the following criteria: (1) irreducible proper fractions; (2) both the numerator and denominator are single-digit numbers; (3) paired fractions share neither a common numerator nor a common denominator; and (4) the magnitude of the fraction pairs cannot be determined based solely on whole number information. The fraction pairs utilized for close distance comparisons were as follows: (5/8 and 4/9); (3/4 and 5/9); (7/8 and 5/9); (1/3 and 2/7). The fraction pairs employed for far distance comparisons included: (5/6 and 1/7); (4/7 and 1/8); (5/8 and 1/9); (6/7 and 2/9). For the non-symbolic stimulus materials, we adopted the methodology from Pomè et al. (2019), making every effort to control for potential confounding factors such as convex hull, surface area^102^. Furthermore, the total area of white and black regions was kept equal across the two sets of dot patterns to ensure that participants could not rely on visual area as a cue during the comparison task.

### Design and procedure

We employed a 2 (symbol format: symbolic, non-symbolic) × 2 (distance: far distance, close distance) within-subjects factorial experimental design. In this study, both symbol format and distance were treated as within-subject variables.

**Fig. 1:**
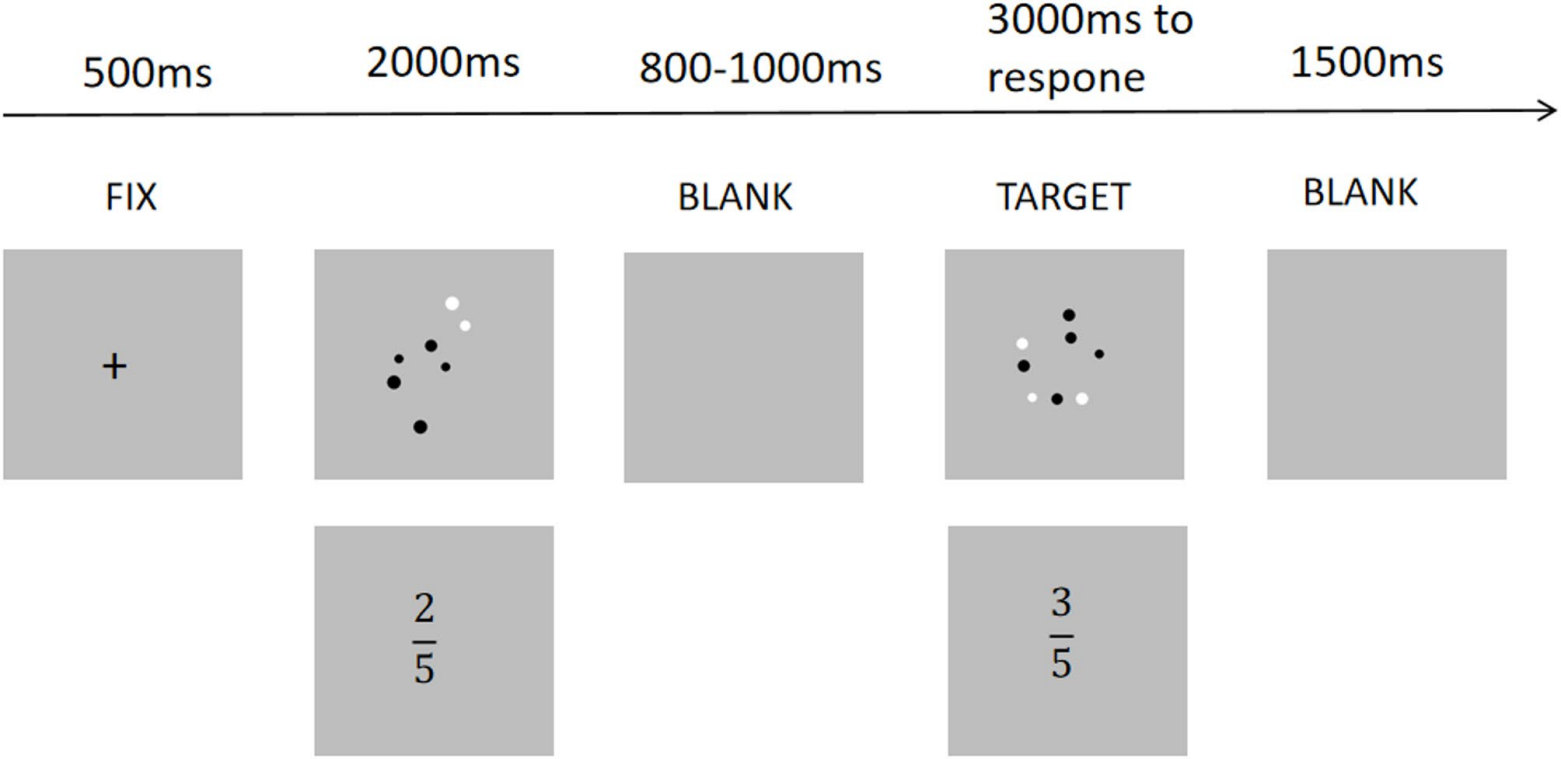
An example of a close-distance trial involving the symbolic and non-symbolic fraction comparison task.

The experiment was conducted in a quiet room, with participants seated approximately 60 centimeters away from the computer screen. The experimental stimuli were presented on a 24-inch monitor using E-Prime 2.0 software, with a resolution of 1920 × 1080 pixels. The background of the screen was gray, while the stimuli consisted of black and white dot arrays. The presentation sequence for each trial of the task is as follows: At the beginning of each trial, a black fixation cross will appear and remain on the screen for 500 milliseconds. Subsequently, a proportional stimulus will be presented, lasting for 2000 milliseconds. Following this, a random blank screen will be displayed for a duration ranging from 800 to 1000 milliseconds. Afterward, the proportional stimulus will reappear, with a maximum duration of up to 3000 milliseconds. Participants are required to respond within this time limit by comparing it with the initially presented proportional stimulus. Finally, a blank screen will be shown for 1500 milliseconds. Then the next trial was presented (Fig. 1).

The stimuli were presented in a sequential order, and participants were required to determine whether the second proportional stimulus was larger or smaller than the first. The experiment consisted of six blocks, with symbolic formats alternating in presentation. Each pair of fractions was displayed in two different sequences (i.e., either the symbolic stimulus was presented first or the non-symbolic stimulus). Each block comprised 120 trials. Furthermore, we ensured balance between left and right key presses during the experimental task. To mitigate participant fatigue, breaks between each block were allowed at participants’ discretion, and blinking was permitted between individual trials.

### EEG data recording

We employed the Neuroscan 4.5 EEG analysis and recording system (Compumedics Neuroscan, Compumedics USA) along with Compumedics Neuroscan Curry 8.0 software (https://www.compumedics.com.au) to record EEG data using a non-invasive electrode cap featuring 64 channels arranged according to the international 10–20 system. The Cz electrode located at the vertex served as an online reference, while the midpoint between Fz and FPz electrodes was grounded. To monitor blink and horizontal eye movements, we recorded vertical eye movements using electrodes positioned above and below the left eye, and horizontal eye movements were captured by two electrodes placed at the outer canthi of both eyes. During the experiment, we utilized a Neuroscan SynAmps 2.0 amplifier for data acquisition in DC mode with a sampling rate of 1000 Hz, ensuring that throughout the entire experiment, scalp impedance for each electrode site remained below 5 kΩ.

### EEG data analysis

The analysis of EEG data was conducted using the EEGLAB 9.0 and ERPLAB 14.0 toolboxes for offline processing. During the offline analysis, the average value from bilateral mastoids was utilized as a reference electrode. To mitigate the impact of artifacts and EEG drift caused by participants’ blinking, horizontal eye movements, and excessive muscle activity during the experiment on the data results, this study employed Independent Component Analysis (ICA).

Based on existing research^103^, this study employed a band-pass filter ranging from 0.01 to 30 Hz. The Temporal Progression for ERP analysis was set from 100 milliseconds prior to the presentation of the target stimulus (as baseline) to 1000 milliseconds following its presentation. Prior to averaging, trials with amplitudes exceeding ± 100 µV were considered artifacts and were automatically excluded. During the artifact rejection process, the rejection rate did not exceed 25%.

In the analysis of ERP data, based on the objectives of this study and previous research findings^70,90^, we selected electrode sites in the left and right parietal regions, including P3, P7, PO3, PO7, and O1 as well as P4, P8, PO4, PO8, and O2. We analyzed the potentially elicited N1 component within a time window of 100 to 150 ms. Additionally, we chose electrode sites in the central and parietal regions of the brain—namely CP1, CP2, POz, P1, P2, CPz, and Pz—to examine the potentially elicited P3 component within a time window of 350 to 450 ms. The mean amplitude values of the N1 and P3 components under different experimental conditions were subjected to a repeated-measures analysis of variance using IBM SPSS Statistics (Version 26.0; https://www.ibm.com/products/spss-statistics) to evaluate the research hypothesis. When the results did not meet the sphericity assumption, the Greenhouse-Geisser method^104^ was employed to correct the degrees of freedom in the *F*-test. For post-hoc multiple comparisons, the Bonferroni method was applied to adjust *p*-values.

### Statement

The study involving human subjects was conducted in accordance with the Declaration of Helsinki. This research received approval from the Ethics Committee of the School of Psychology at Guizhou Normal University (Approval Code: GZNUPSY.N.202501E [0024]; Date: 2025.1.15). Prior to participation, all individuals provided informed consent by signing appropriate forms. All methodologies were executed in compliance with relevant guidelines and regulations.

## Results

### Behaviors results

#### Accuracy

Based on previous study^70^, we conducted a repeated measurement variance analysis on the accuracy rates of participants in processing fractions, employing a 2 (symbol format: symbolic, non-symbolic) × 2 (distance level: far distance, close distance) design. In this analysis, both symbol format and distance level were treated as within-subject variables. The results indicate that the main effect of distance level is significant, *F* (1, 28) = 33.763, *p* < 0.001, η^2^_*p*_ = 0.547. Specifically, the accuracy in the far-distance condition (0.977 ± 0.003, M ± SE, same below) was significantly higher than that in the close-distance condition (0.918 ± 0.010). However, the main effect of symbol format did not reach statistical significance, *F* (1, 28) = 2.796, *p* = 0.106.

**Fig. 2:**
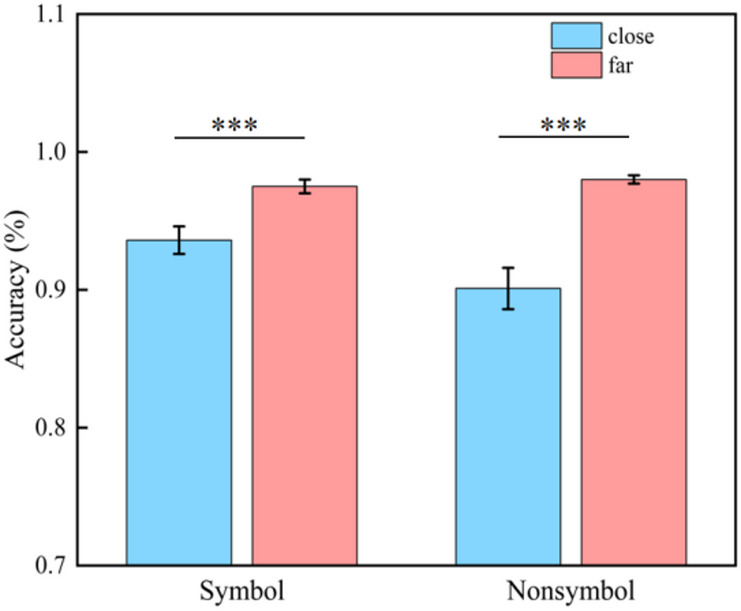
The proportions of correct trials for both non-symbolic and symbolic conditions are presented, with error bars representing ± 1 standard error (SE). Close, close-distance fractional pairs; Far, far-distance fractional pairs. * indicates *p* < 0.05, ** indicates *p* < 0.01, and *** indicates *p* < 0.001. The meaning of the asterisks in all subsequent figures of this study is consistent with that in this figure.

Importantly, the interaction between symbol format and distance level is significant, *F* (1, 28) = 10.647, *p* = 0.003, η^2^_*p*_ = 0.275. Further analysis of simple effects reveals that the symbol format, participants’ accuracy in the far distance condition (0.975 ± 0.005) is significantly higher than in the close distance condition (0.936 ± 0.010), *F* (1, 28) = 28.826, *p* < 0.001, η^2^_*p*_ = 0.507. Moreover, within the non-symbol format, the accuracy under the far distance condition (0.980 ± 0.003) is also higher than the accuracy under the close distance condition (0.901 ± 0.015), *F* (1, 28) = 28.209, *p* < 0.001, η^2^_*p*_ = 0.502 (Fig. 2).

### Event-related potentials

#### N1 (150–200 ms)

The repeated measurement variance analysis conducted on N1 component with a 2 (symbol format: symbolic, non-symbolic) × 2 (region: left region, right region) design revealed a significant main effect of symbol format, *F* (1, 28) = 32.663, *p* < 0.001, η^2^_*p*_ = 0.538. The amplitude of the N1 component evoked by non-symbolic fractions (−1.939 ± 0.561 µV) was more negative than that evoked by symbolic fractions (0.511 ± 0.481 µV).

**Fig. 3:**
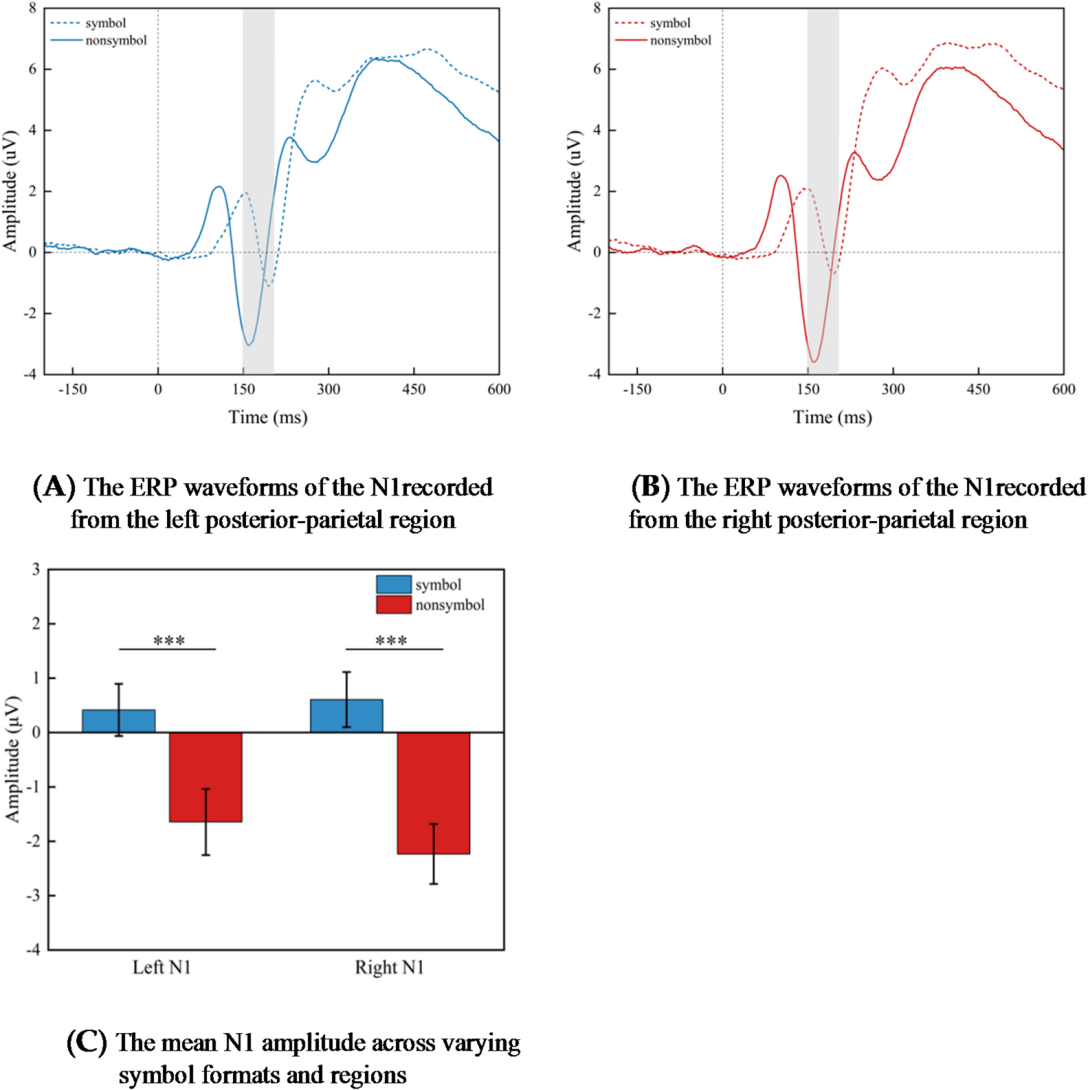
The mean N1 amplitude (µV) for pairs presented in both symbolic and non-symbolic formats is displayed. Graphs A and B illustrate the average amplitude recorded from left and right posterior-parietal electrodes over time, with the N1 time window highlighted in gray. Graph C illustrates the average amplitude recorded at N1. The error bars represent ± 1 standard error (SE). Symbolic, symbolic numerical formats; Nonsymbolic, non-symbolic numerical formats.

Importantly, there were significant interactions between format and region, *F* (1, 28) = 6.820, *p* = 0.014, η^2^_*p*_ = 0.196. Further analysis of simple effects reveals that within the left parietal cluster, the difference in N1 component amplitude evoked by symbolic and non-symbolic fractions is significant, *F* (1, 28) = 21.173, *p* = 0.000, η^2^_*p*_ = 0.431. The N1 amplitude evoked by non-symbolic fractions (−1.645 ± 0.609 µV) is more negative than that induced by symbolic fractions (0.416 ± 0.479 µV). Moreover, we found that within the right parietal cluster, the N1 amplitude evoked by non-symbolic fractions was significantly more negative (−2.234 ± 0.551 µV) compared to that evoked by symbolic fractions (0.606 ± 0.507 µV), *F* (1, 28) = 38.127, *p* = 0.000, η^2^_*p*_ = 0.577 (Fig. 3).

#### P3 (350–450 ms)

The repeated measurement variance analysis conducted on P3 component with a 2 (symbol format: symbolic, non-symbolic) × 2 (distance level: close distance, far distance) design revealed a significant main effect of distance level, *F* (1, 28) = 8.402, *p* = 0.007, η^2^_*p*_ = 0.231. The amplitude of the P3 component evoked by far distance (6.408 ± 0.604 µV) was greater than that evoked by close distance (5.580 ± 0.734 µV). But there were no main effects of format, *F* (1, 28) = 0.204, *p* = 0.655.

**Fig. 4:**
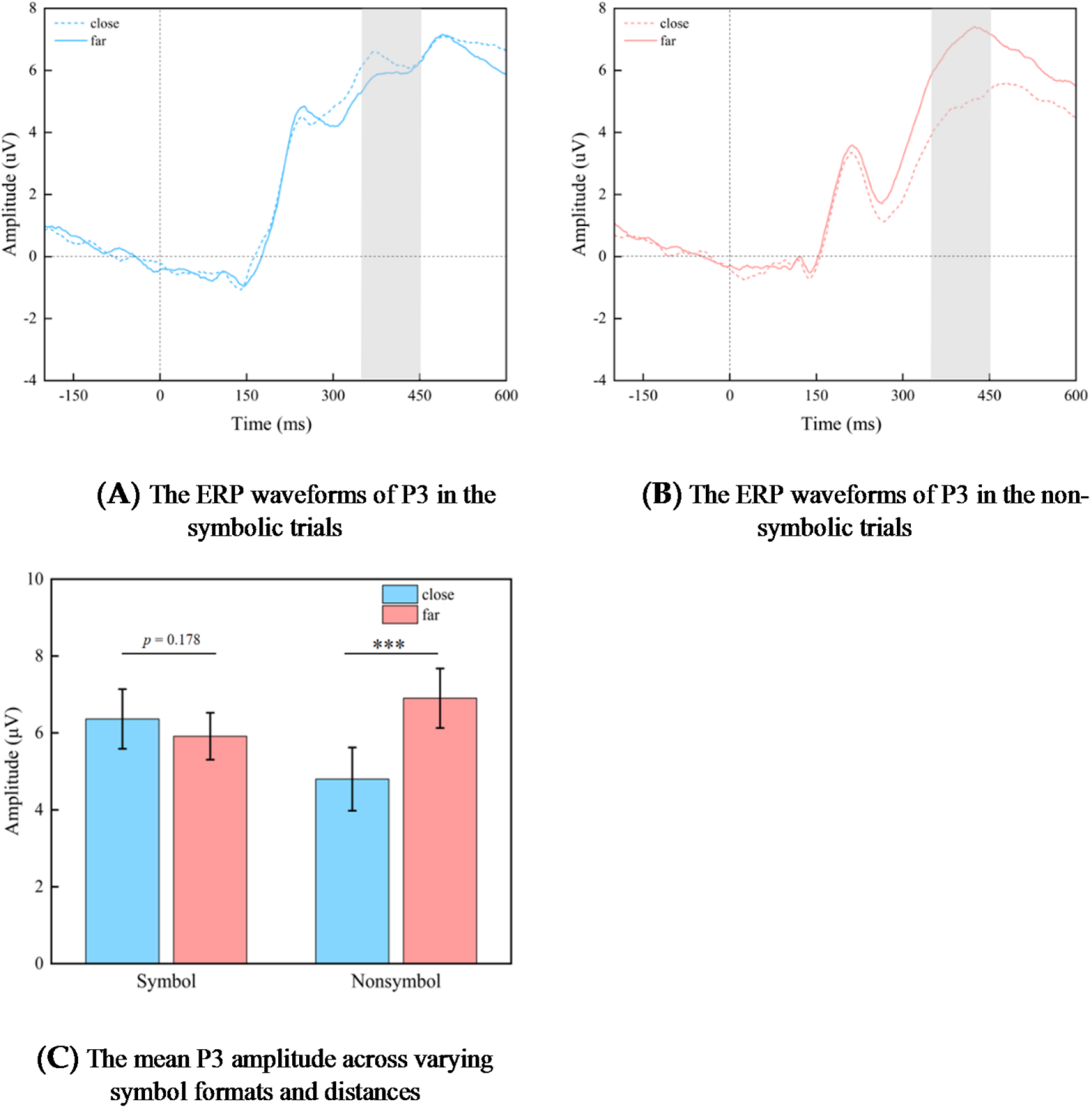
The mean P3 amplitude (µV) for pairs exhibiting close and far distance magnitude values in both symbolic and non-symbolic formats is presented. Graphs A and B illustrate the average amplitude recorded from posterior central electrodes over time, with the P3 time window highlighted in gray. Graph C illustrates the average amplitude recorded at P3. The error bars represent ± 1 standard error (SE). Close, close-distance fractional pairs; Far, far-distance fractional pairs.

More importantly, there was an interaction between format and distance level on P3 amplitude, *F* (1, 28) = 47.047, *p* = 0.000, η^2^_*p*_ = 0.627. Further analysis of simple effects reveals that within the symbolic format, the difference in P3 amplitudes evoked by far distance (5.914 ± 0.610 µV) and close distance (6.362 ± 0.775 µV) stimuli was not significant, *F* (1, 28) = 1.911, *p* = 0.178. However, there was a significant effect of distance with non-symbolic fractions, *F* (1, 28) = 34.699, *p* = 0.000, η^2^_*p*_ = 0.553. The P3 amplitude evoked by far distance conditions (6.903 ± 0.774 µV) is significantly greater than that observed under close distance conditions (4.799 ± 0.823 µV) (Fig. 4).

## Discussion

The relationship between the processing of symbolic and non-symbolic fraction has long been a significant research topic in the field of numerical cognition. The study investigates whether symbolic and non-symbolic fractions are dependent of notion in the process of numerical processing, utilizing behavioral experiments and electrophysiology techniques. The results of the accuracy data indicate that we observed a numerical distance effect in the processing of both symbolic and non-symbolic fractions. This finding suggests that both formats of fractions can be represented holistically. This conclusion is consistent with previous study findings^78,80,105–108^. Although this finding is consistent with previous research results, the outcomes of electrophysiology experiments can provide a deeper insight into the underlying mechanisms involved in the processing of symbolic and non-symbolic fractions compared to behavioral data. The ERP data results indicate that, compared to symbolic fractions, non-symbolic fractions evoked a significantly more negative amplitude of the N1 component. Even more importantly, we found that within the non-symbolic format, the amplitude of the P3 component evoked by far distance was significantly greater than that evoked by close distance. However, this phenomenon was not observed within the symbolic format. The findings of this study are consistent with prior research^70^, indicating that the ratio of discrete (dot array) configurations is similar to that of continuous (vertical lines) representations, both of which can reliably perform numerical representation. Previous research has also found that there are differences in cognitive processing between continuous representation and discretization or discrete representation^74,98^. However, Fabbri et al. (2012) discovered in a non-symbolic fraction study that ratio judgments of non-symbolic fractions (i.e., dot arrays) are influenced by numerical information; nevertheless, this influence does not alter the representation strategy employed for non-symbolic fractions^109^. These findings align with those of the current study. Overall, the magnitude of non-symbolic fractions can be processed automatically and holistically.

In our ERP data results, we observed a significant effect of symbolic format within the left and right parietal clusters. Specifically, non-symbolic fractions elicited more negative N1 component amplitudes compared to symbolic fractions. During the cognitive processing stage, the neural activity associated with non-symbolic fractions exhibits a higher level of activation compared to that of symbolic fractions. Numerous EEG studies have indicated that the N1 component is associated with the sensory discrimination processing of symbolic stimuli^92,110,111^. The study found a significant difference in the amplitude of the N1 component evoked by symbolic fractions and non-symbolic fractions. This indicates that participants employed distinct sensory discrimination processing strategies when dealing with these two formats of fractions. Based on this result, we propose that the increased familiarity with symbolic fractions may stem from individuals’ gradual training and learning since they formally began their education. Consequently, compared to non-symbolic fractions, symbolic fractions are cognitively more familiar and comprehensible, making them easier to discern. The N1 component is also believed to vary in response to changes in continuous sensory cues, such as the integrated surface area and density of dot arrays^58,93,112,113^. Therefore, we can also speculate that, compared to symbolic fractions, individuals may process sensory cues such as the surface area of dot aggregates in non-symbolic fractions. This processing difference could potentially lead to variations in the N1 amplitude between the two formats of fractions. In summary, there is an independence in sensory discrimination between symbolic fractions and non-symbolic fractions.

In the P3 component, we observed an interaction between symbol format and distance level. Specifically, non-symbolic fractions elicited a significantly greater P3 amplitude under far distance compared to close distance, whereas symbolic fractions did not exhibit this phenomenon. Previous studies have demonstrated that the P3 component is linked to the representation and processing of numerical magnitude^90,92,114^. The results indicate that non-symbolic fractions and symbolic fractions may exhibit notion dependent characteristics during numerical processing. This finding supports the encoding complex hypothesis proposed by Campbell et al.^71–73^. According to this theory, numbers are not represented in an abstract form but rather rely on independent modality-specific numerical coding. Therefore, it can be inferred that symbolic fractions and non-symbolic fractions are represented through two distinct magnitude processing systems. The results of the ERP study indicate that non-symbolic fractions are processed using a holistic representation strategy. This finding is consistent with previous research outcomes^83,84,105^. Regarding symbolic fractions, previous research has indicated that when processing fractions, participants utilize both componential and holistic processing strategies^80,90,106^. Meert et al. (2010b) found in an experimental task involving adult participants that the representation of fraction magnitude is hybrid rather than purely holistic. In summary, symbolic and non-symbolic fractions employ different processing strategies^106^.

According to the research conducted by Kallai and Tzelgov^115^, the magnitude of fractions is likely perceived initially as entities less than 1, thereby facilitating holistic processing. However, employing a component-based processing strategy to execute tasks appears to be more efficient, whereas holistic processing may be subject to interference and inhibition. The representation of fractions may employ a hybrid representation strategy, to some extent indicating that it is based on the integer symbol system^116^, rather than having an innate representation mechanism, and will be influenced by the whole number strategy. That is to say, during the comparison of fractions, there will be a “whole number bias” phenomenon. Participants will judge the size of the fraction value based on the magnitudes of the numerator and denominator, and will also flexibly adopt appropriate processing strategies (e.g., holistic processing strategy) according to the specific task context. Therefore, during the process of fraction processing, neither the component processing strategy nor the holistic processing strategy predominates. However, the coexistence of component processing and holistic processing may obscure the representation of fractional magnitudes, leading to a lack of distance effect in the P3 component under symbolic fraction conditions. The study suggesting that in non-symbolic fraction tasks, proportion judgments can also be influenced by whole number strategies^74^. For non-symbolic fractions, whole number information affects individuals’ proportion judgments; however, individuals still employ a holistic representation strategy when processing non-symbolic fractions^109^. We argue that, from the perspective of human development, prior to the emergence of symbolic representation systems, individuals primarily realized numerical representations through physical means, such as dot arrays. Therefore, individuals possess two distinctly different numerical systems—physical representation and symbolic representation. This divergence results in the adoption of varying representational strategies during the processing of non-symbolic fractions and symbolic fractions. For the physical representation of non-symbolic fractions, some studies suggest that it is based on the RPS for representing and processing proportions^83^. Currently, empirical research on RPS remains relatively limited. Future studies could yield additional evidence through behavioral experiments and brain imaging to substantiate the existence of the RPS. In summary, the current data provide evidence for the existence of distinct numerical systems associated with symbolic fractions and non-symbolic fractions.

This study has certain limitations. Although the experimental stimuli effectively prevented participants from adopting a simplistic strategy—completing the task solely by comparing the integer parts of the fractions—future research could enhance methodological rigor by incorporating consistency conditions (where whole number information aligns with fractional magnitude) and inconsistency conditions (where whole number information contradicts fractional magnitude). However, such manipulations may affect the discriminability of fraction pair distances and the total number of experimental items. Therefore, future studies should strive to balance these two methodological factors. Regarding the surface area balance of dot arrays in non-symbolic stimulus materials, future experiments can implement a design in which, in half of the consistent trials, the group with the larger numerical magnitude occupies a greater display area; whereas in the remaining half of the inconsistent trials, the same group occupies a relatively smaller display area. Furthermore, future research could investigate the neuroimaging-level spatial brain structures involved in processing symbolic and non-symbolic fractions. Additionally, it may examine age-related differences in the processing of symbolic and non-symbolic fractions across the lifespan. Such investigations would contribute to a deeper understanding of the underlying mechanisms and theoretical significance of symbolic and non-symbolic numerical processing.

## Conclusions

Existing behavioral and neuroimaging studies indicate a debate regarding symbolic and non-symbolic representations: whether non-symbolic representations serve as the foundation for the mapping of symbolic representations, or whether the non-symbolic numerical system and precise symbolic numerical system develop as two independent representational systems. In the present study, we employed electrophysiological techniques to investigate the temporal processes involved in the numerical processing of symbolic and non-symbolic fractions. In the present study, we employed electrophysiological techniques to investigate the temporal processes involved in the numerical processing of symbolic and non-symbolic fractions. Our findings suggest that individuals have access to two fundamentally distinct numerical systems: a non-symbolic representation system and a symbolic number system. During the period of formal education, educators should enhance students’ sensitivity to symbolic meanings through continuous training, thereby aiding their understanding of the significant role that numerical symbols play in magnitude processing. Additionally, fostering students’ strategies for handling fractions can effectively improve their mathematical performance. To sum up, we believe that the magnitude processing of fractions is based on the notation dependent representation^117–120^.

## Data Availability

The data supporting the conclusions of this study are available from the corresponding author upon reasonable request.
